# Non response, incomplete and inconsistent responses to self-administered health-related quality of life measures in the general population: patterns, determinants and impact on the validity of estimates — a population-based study in France using the MOS SF-36

**DOI:** 10.1186/1477-7525-11-44

**Published:** 2013-03-13

**Authors:** Joel Coste, Laurent Quinquis, Etienne Audureau, Jacques Pouchot

**Affiliations:** 1 Biostatistics and Epidemiology Unit, Assistance Publique-Hôpitaux de Paris, Hôtel Dieu, Paris, France; 2 Research unit APEMAC, EA 4360, Nancy-Université, Université Paris-Descartes, Université Metz Paul Verlaine, Paris, France; 3 Department of Internal Medicine, Assistance Publique-Hôpitaux de Paris, Hôpital Européen Georges Pompidou, Paris, France

**Keywords:** Quality of life, SF-36, Missing items, Missing forms, Determinants, Bias, Imputation

## Abstract

**Background:**

Health-related quality of life (HRQoL) measures are increasingly used in the general population. However, little is known about patterns and determinants of unanswered or unusable questionnaires and their consequences on estimates of HRQoL.

**Methods:**

The 2003 Decennial Health Survey collected socio-demographic and health information, including HRQoL, for 30,782 adults representative of the French population. The pattern, determinants and impact on estimate validity of non, incomplete and inconsistent responses to the SF-36 questionnaire were determined. For this, phi coefficients, polytomous logistic regression models and multiple imputation methods were used.

**Results:**

Only 48% of the subjects eligible for the HRQoL measurement provided a complete and consistent SF-36 questionnaire. Three patterns of non-response and five of partial (incomplete or inconsistent) response were identified, sharing largely similar socio-demographic profiles (higher age, lower educational level and economic status, foreign background, and isolated). The consequences of non and partial responses on HRQoL estimates were large in several groups of subjects although these biases ran in opposite directions and partially neutralized each other.

**Conclusions:**

When measuring HRQoL in the general population, missing and inconsistent data are frequent, especially in elderly, educationally and socio-economically deprived, foreign and isolated groups. Methods for handling missing data are required to correct for potentially the associated and serious selection and non-differential information biases in studies targeting or investigating these groups.

## Introduction

Health-related quality-of-life (HRQoL) measures are increasingly used, complementary to mortality and morbidity indicators, to assess health status in the general population e.g. [1-3]. However, little is known about patterns and determinants of non response and unusable questionnaires and their effects on estimates of HRQoL in the population setting. As with other health status measures, both non responses and incomplete responses reduce the statistical power of studies (by reducing sample size). They may also cause biases if non- or partial-respondents differ from respondents as concerns HRQoL or its determinants or confounders.

The problem of missing measures in HRQoL studies has mostly been addressed in clinical research and separately for forms (questionnaires as a whole) and items [4,5]. Few studies have considered this issue in the general population [6-9]. In addition, in studies addressing this problem in the general population, non response to self-administered HRQoL measures was not distinguished from non response to other health information. Similarly, little attention has been paid to inconsistent responses to HRQoL items and their determinants, although in some cases the algorithms for scoring questionnaires take them into account [10].

The present empirical study extends and generalizes an approach developed in two previous articles [9,11]. It used data from a large population-based survey to successively investigate the pattern, the determinants and the impact on the validity of estimates of HRQoL of non response (missing forms), incomplete response (missing items of various patterns) and inconsistent response to the HRQoL measure used: the Medical Outcomes Study (MOS) 36-item short-form (SF-36) questionnaire.

The research questions addressed in this paper are as follows:

1) Can a general pattern of non- and partial response be defined? Can a mutually exclusive categorization scheme be proposed, which would be conceptually sound and empirically substantiated?

2) Do the categories identified in response to our first research question have specific determinants? Are the processes of non- and partial response affected by particular socioeconomic variables, health behaviors, chronic conditions or age?

3) What is the impact of non- and partial response on the validity of estimates of HRQoL? Are the effects similar for each process? Is it necessary to correct for biases, either self-selection or (non differential) information?

The study perspective was that of a researcher confronted by both missing and inconsistent forms, and missing items in a HRQoL study, willing to investigate the resulting biases and control for them. This situation is not uncommon in epidemiological practice, especially when general or community populations are concerned.

## Methods

### Population sample and collected data

The Decennial Health Survey is a national survey of households, representative of the French population, performed on a 10-year basis since 1970 [12]. Its sampling is clustered and stratified (on region and size of urban unit) and random, all individuals in the households selected being included in the survey. In the first step, specifically trained interviewers collected, during home visits, socio-demographic characteristics and information on health status. Present and past chronic morbidities were addressed and recorded by the interviewers and were further coded using the ICD-10 (international classification of diseases, 10th revision). During this visit, several self-administered questionnaires, including the SF-36 were given to the subjects. These questionnaires were collected during a second home visit 2 months later.

### Health-related quality of life measurement

The French SF-36 questionnaire [13,14] (version 1.3) used in the Decennial Health Survey was developed and validated as part of the International Quality of Life Assessment (IQOLA) project [15]. It is made up of 35 questions divided into eight dimensions: physical functioning (PF1 to PF10), role limitations relating to physical health (RP1 to RP4), bodily pain (BP1 and BP2), general health perceptions (GH1 to GH5), vitality (VT1 to VT4), social functioning (SF1 and SF2), role limitation relating to mental health (RE1 to RE3), and mental health (MH1 to MH5). One additional item assesses health transition (HT). Each question is rated on an ordinal scale with 2 to 6 categories. As recommended by Ware et al. [10], the score of each dimension was computed if at least half of the items of the related dimension were available (“half item rule”) (this rule is equivalent to imputing a missing item with the mean value of the other non missing items of the same dimension, a method known as “personal mean score” [PMS] imputation); the score of the dimension is the sum of the item scores further normalized to range from 0 to 100, with higher values representing better perceived QoL. This self-administered questionnaire can be completed rapidly (5–10 min) and is well-adapted for studies in general populations.

### Statistical analysis

#### General strategy and steps of the analysis

In accordance with our objectives, the statistical analysis was based on a three-step methodology. It involved successively identifying existing patterns of non- and partial response, exploring the potential determinants of these patterns, and finally assessing their impact on the validity of estimates of HRQoL.

#### Patterns of non- and partial responses

The first step of the approach was to identify patterns of non- and partial responses. To do so, we hypothesized that patterns of response result from three main factors:

(i) Non response linked to the occurrence of missing forms;

(ii) Partial response linked to the occurrence of missing items;

(iii) Inconsistent response linked to the occurrence of inconsistency between items.

Non responders were further categorized according to the three main reasons recorded for the form being missing:

(i) unable to fill-out the SF-36 questionnaire, due mainly to cognitive or physical deficiencies;

(ii) subjects absent at the 2-month visit, a significant proportion of which may be subjects implicitly refusing further inclusion in the survey;

(iii) declining to complete the SF-36 (which can be considered to be specific disinterest in, or refusal to contribute to, the HRQoL measure).

For responders, missing items were ascertained and inconsistent responses were identified following the rules described by Ware et al. [10], for 15 pairs of items. For example, subjects replying that they “walk one block” (PF9) with difficulty but also that they performed “vigorous activities” (PF1) without difficulty are considered “inconsistent” (see Table 1 for the list of inconsistencies). A 51 (36 +15) × 51 matrix of phi-coefficient was then constructed to identify patterns of partial and inconsistent responses (the phi-coefficient is a correlation coefficient for two dichotomous variables).

**Table 1:** Scales, items of the SF-36 questionnaire and proportion of missingness and inconsistencies of response

**Scales / Items / Inconsistencies**	**Scores (ordinal Likert)**	**Missing response n (%)**	**Inconsistent response n (%)**
**PF (Physical functioning )**			
PF1 Vigorous activities	1 to 3	696 (3.0)	
PF2 Moderate activities	1 to 3	718 (3.1)	
PF3 Lift, carry groceries	1 to 3	745 (3.2)	
PF4 Climb several flights	1 to 3	806 (3.5)	
PF5 Climb one flight	1 to 3	1104 (4.8)	
PF6 Bend, kneel	1 to 3	746 (3.2)	
PF7 Walk >1 km	1 to 3	703 (3.1)	
PF8 Walk several blocks	1 to 3	1020 (4.4)	
PF9 Walk one block	1 to 3	1212 (5.3)	
PF10 Bathe, dress	1 to 3	629 (2.7)	
Inconsistency PF1- PF9			487 (2.1)
Inconsistency PF1- PF10			615 (2.7)
Inconsistency PF2- PF9			448 (1.9)
Inconsistency PF2- PF10			505 (2.2)
Inconsistency PF4- PF9			405 (1.8)
Inconsistency PF4- PF10			524 (2.3)
Inconsistency PF7- PF9			288 (1.3)
Inconsistency PF7- PF10			434 (1.9)
**RP (Role limitations relating to physical health )**			
RP1 Cut down time working	1 to 2	722 (3.1)	
RP2 Accomplished less	1 to 2	722 (3.1)	
RP3 Limited in type of work	1 to 2	722 (3.1)	
RP4 Difficulty performing work	1 to 2	722 (3.1)	
**BP (Bodily pain)**			
BP1 Intensity of bodily pain	1 to 6	547 (2.4)	
BP2 Extent that pain interferes with work	1 to 6	547 (2.4)	
Inconsistency BP1- BP2			14 (0.1)
**GH (General health perceptions)**			
GH1 General health	1 to 5	1450 (6.3)	
GH2 Get sick more easily	1 to 5	1451 (6.3)	
GH3 As healthy as anybody	1 to 5	1365 (5.9)	
GH4 Expect health to get worse	1 to 5	1390 (6.0)	
GH5 Health is excellent	1 to 5	1533 (6.7)	
Inconsistency GH1- GH5			23 (0.1)
**VT (Vitality)**			
VT1 Full of life	1 to 6	1275 (5.5)	
VT2 Energetic	1 to 6	1275 (5.5)	
VT3 Worn out	1 to 6	1246 (5.4)	
VT4 Tired	1 to 6	906 (3.9)	
VT2- VT3			543 (2.4)
VT1- VT4			536 (2.3)

#### Identification of factors associated with non- and partial responses

The second step of the approach was to identify determinants associated with patterns of non- and partial responses using polytomous (nominal) logistic regression models. The responder subjects without any missing items or inconsistency, referred to as “complete and consistent responders”, formed the reference category for this analysis. The mutually exclusive patterns of non- and partial response identified in the first step were the other categories of the nominal variable. Regression models were constructed to identify the determinants of the various non- and partial response patterns. The models were constructed in four successive stages. First, socioeconomic variables (education, marital status, occupational status, income), geographic origin and foreign background were included in the model. Second, health behaviors (smoking, alcohol dependency according to Cage score [16]), third, the most frequent chronic conditions and Charlson [17] and Elixhauser [18] comorbidity scores (unweighted scores computed using ICD-10 codes of present chronic morbidities, ranging 0 to 17 and 0 to 30 respectively), and fourth, age, were added to significant predictors identified in the previous steps (age was assessed as a “residual effect” after the intermediary effects of comorbidities were taken into account).

At each stage of the model construction, a backward elimination procedure was used to select the significant independent variables and two-way interactions terms (p < 0.05) to be kept in the model. Due to several interactions between sex and identified predictors, models were eventually constructed (and are reported) for men and women separately. Because of the probable correlation between subjects from the same households, we tested whether such a clustering effect may affect the estimates of predictors by using random intercept models that allow intercepts to differ between households (multilevel analysis). Results from this sensitivity analysis were comparable to those from standard fixed effects models (monolevel analysis). Consequently, only results from standard fixed effects models are reported here. These analyses were performed using STATA 11.0 (multilevel modeling, xtmelogit, StataCorp, College Station, TX) and SAS 9.2 (standard fixed effects modeling, PROC LOGISTIC, SAS Inst., Cary, NC) software packages.

#### Assessment of the effects on the validity of estimates of HRQoL, and quantification of biases due to non- and partial responses

The third step of the approach was to assess the magnitude of the bias in HRQoL estimation due to non- and partial responses. Firstly, mean values for each dimension of the SF-36 were computed according to recommended “standard rules” [10], including:

(i) recoding inconsistent responses as “missing”;

(ii) using the “half item rule” described above for computing a dimension score (these rules are applicable to *partial responders only*). The application of these rules leads to a large proportion of the sample being excluded from computations.

Secondly, these mean values were compared to those for all subjects after missing scores (for many) were imputed using relevant available (non missing) information. In the case of missing items, the information used was other non missing items of the same dimension, and other dimensions of the SF-36 together with socio-demographic and morbidity data. In the case of missing forms the information used was only socio-demographic and morbidity data.

We used the multiple imputation procedure in SAS statistical software (SAS Proc MI, SAS Inst., Cary, NC) to impute missing data. Variables included in the imputation models were those used in the polytomic logistic regression (socio-demographic parameters, comorbidity and SF36 dimensions). Following the recommendations of Graham [19], 20 datasets were created using the Monte Carlo Markov Chain (MCMC) with a single chain. The starting value for the chain was computed from the Expectation-Maximization (EM) algorithm. A total run length of 200 iterations was performed for computation of the initial values and 100 iterations were run between imputations. This method is widely used and has been shown to be one of best for dealing with missing items in quality of life questionnaires [11]. Its accuracy makes it possible to use scores estimated this way as a “gold standard” to compare scores obtained using standard rules.

## Results

Among 30,996 adult subjects eligible for this Decennial Health Survey, 214 (0.7%) were incapable of responding to any health-related question in French and were excluded from this study. Demographic and socio-economic characteristics, alcohol and smoking status, and Elixhauser and Charlson scores of those 30,782 eligible for the HRQoL measurement are presented in Table 2. The morbidities and conditions most frequently reported by these subjects are presented in Additional file 1 Table S1.

**Table 2:** Characteristics of subjects eligible for HRQoL measurement (n = 30,996)

	
**Age**	
<60	22922 (74.0)
60–64	1846 (6.0)
65–69	1875 (6.0)
70–74	1721 (5.6)
75–79	1294 (4.2)
80–84	886 (2.9)
>85	452 (1.5)
**Gender**	
Men	14798 (47.7)
Women	16198 (52.3)
**Region of residence**	
Paris region	7005 (22.6)
Eastern Parisian Basin	4608 (14.9)
Western Parisian Basin	1979 (6.4)
West	3175 (10.2)
East	2158 (7.0)
Mediterranean Basin	4115 (13.3)
North	3029 (9.8)
South-East	2393 (7.7)
South-West	2534 (8.2)
**Education**	
No diploma	2637 (8.5)
Primary school	7137 (23.0)
Lower secondary level	2274 (7.3)
Intermediate Secondary level	8518 (27.5)
Upper Secondary level	3286 (10.6)
Lower tertiary level	5241 (16.9)
Upper tertiary level	1903 (6.1)
**Marital status**	
Married/in couple	17455 (56.3)
Single	8962 (28.9)
Divorced/separated	2276 (7.3)
Widowed	2303 (7.4)
**Geographic origin and foreign background**	
Metropolitan France	26845 (86.6)
Foreign	4035 (13.0)
**Occupation**	
Active	16534 (53.3)
Inactive-retired	14462 (46.7)
**Mean income (10,000 €/year per household unit) (SD)**	1.63 (1.12)
**Smoking Status**	
Non-smoker	16822 (54.3)
Light smoker (1–9 cig/day)	2035 (6.6)
Moderate consumption smoker (10–9 cig/day)	2439 (7.9)
Heavy smoker (≥ 20 cig/day)	1886 (6.1)
**Alcohol dependence (Cage Score ≥2)**	1624 (5.2)
**Elixhauser score**	
0	26395 (85.8)
1–2	4315 (14.0)
≥3	53 (0.2)
**Charlson score**	
0	22294 (72.4)
1–2	8141 (26.4)
≥3	328 (1.1)

Figures are numbers (percentages) unless stated otherwise.

### Patterns of non- and partial responses

**Figure 1:**
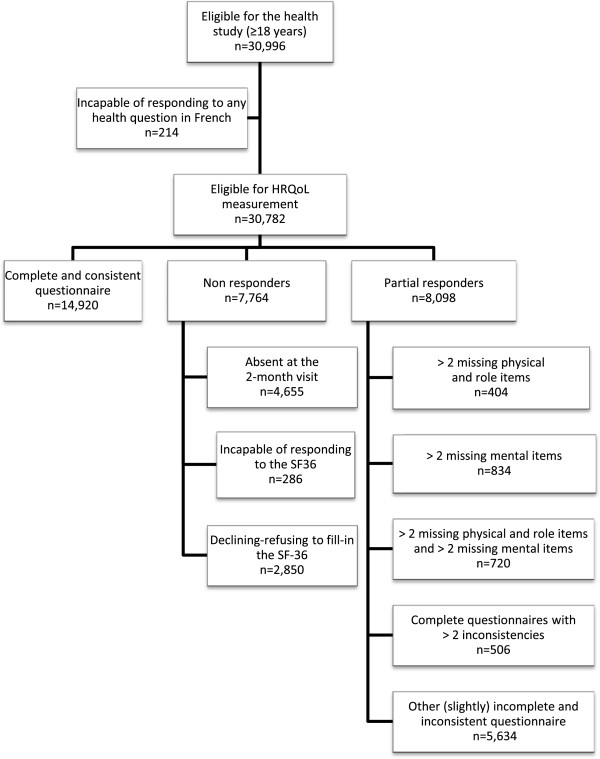
Flowchart of the study design and data analysis strategy.

SF-36 forms were obtained for 23,018 subjects (75% of eligible subjects): 4,655 subjects were absent at the 2-month visit, 286 were found to be incapable of filling-in the SF36 due to physical or cognitive deficits, and 2,850 declined to fill-in the questionnaire (Figure 1).

Only 14,920 subjects (65% of responders and 48% of those eligible for the HRQoL measurement) provided a complete and consistent SF-36 questionnaire (7,105 or 66% of male responders and 7,815 or 63% of female responders).

The proportions of individual missing items from the SF-36 and of inconsistencies for the 15 pairs of items are given in Table 1. Strong relationships (phi-coefficient > 0.60) were observed between (i) missingness to questions related to physical and role dimensions (physical functioning, role–physical, role–emotional), (ii) missingness to questions related to other, mostly “mental”, dimensions (mental health, vitality, bodily pain, general health and social functioning), and (iii) inconstancies concerning any of the pairs of items 1–8 (PF items) and 11–12 (VT items). This observed pattern of partial responses was partly mechanical (i.e., inconsistencies are more likely when items are not missing) but also suggested *three distinct processes* of non-optimal response: inconsistent, incomplete for physical and role items, and incomplete for other, mental, items.

These results led us to consider five categories of partial responders: 1) those providing a consistent but incomplete questionnaire with >2 missing items but only in “physical and role dimensions” (as defined above) (n = 404, 2% of responders); 2) those providing a consistent but incomplete questionnaire with >2 missing items only in other (mostly mental) dimensions (n = 834, 4% of responders); 3) those providing a consistent but incomplete questionnaire with >2 missing items in physical and role dimensions and >2 missing items in other (mostly mental) dimensions (n = 720, 3% of responders); 4) those providing a complete but inconsistent questionnaire with >2 inconsistencies (n = 506, 2% of responders); and 5) others (n = 5,634, 24% of responders) i.e. those providing an only slightly incomplete (≤2 missing items) or inconsistent questionnaire (≤2 inconsistencies) (Figure 1). If we add these five categories of partial responders to the three categories of non responders (see above), we obtain eight patterns of non- and partial responses which can be compared with complete and consistent response.

### Factors associated with non- and partial responses

Several socioeconomic factors were strongly associated with non- and partial responses (Table 3): lower educational level (with a “dose–response” relationship for most categories of non- and partial responses, especially in men), occupation (being economically active), foreign background in men and, to a lesser extent, low income in women. Region of residence (Paris region, Mediterranean basin, South-West) in men and being single, divorced or widowed were also associated with several non-response categories in men and women. Morbidity was quantitatively and qualitatively associated with non- and partial responses. A higher Elixhauser score (rather than Charlson score, data not shown) was found to be associated with most categories of partial and non-response in both sexes, with the exception of being absent which was negatively associated with comorbidity scores. Some of the most frequent morbidities and conditions tested for association with partial and non-response to the SF-36 were indeed associated: diabetes (in both sexes), bilateral blindness (in men), and visual deficiency (in women). Interestingly, a similar number of conditions were associated with a decreased likelihood of partial and non response: hypertension and migraine, and to a lesser extent and less consistently, sleeping disorders (in women) and anxiety disorders and chronic pulmonary diseases. In women, paralysis was associated both with an increased likelihood of non-response (due to being more likely to be absent or incapable of responding) and with a decreased likelihood of inconsistent and partial responses (especially to questions related to physical and role dimensions). Finally, increasing age over 50 years old was found to be very strongly, and independently, associated with being incapable, or not willing, to fill-in the SF-36 in both sexes, and being absent and providing partial responses in women. Belonging to the youngest age category (18–25 years) was only associated with an increased likelihood of being absent.

**Table 3:** Factors associated with non- and partial responses

**A**		**NON RESPONDERS**			**PARTIAL RESPONDERS**				
	**Complete and consistent (Reference)**	**Absent at the 2-month visit**	**Incapable of responding**	**Not willing to fill-in**	**> 2 missing physical and role items**	**> 2 missing mental items**	**> 2 missing physical and role items and > 2 missing mental items**	**Complete questionnaires with > 2 inconsistencies**	**Other (slightly) incomplete and inconsistent questionnaire**
		**OR (95% CI)**	**OR (95% CI)**	**OR (95% CI)**	**OR (95% CI)**	**OR (95% CI)**	**OR (95% CI)**	**OR (95% CI)**	**OR (95% CI)**
**Age**									
18-24	…	**1.3 (1.1-1.6)**	0.3 (0.1-1.2)	0.9 (0.7-1.1)	1.1 (0.5-2.5)	0.7 (0.4-1.2)	0.5 (0.2-1.1)	1.4 (0.9-2.2)	0.9 (0.7-1.1)
25-39		1.0	1.0	1.0	1.0	1.0	1.0	1.0	1.0
40-49		1.0 (0.9-1.2)	1.8 (0.9-3.7)	1.0 (0.8-1.2)	1.8 (1.0-3.3)	1.3 (0.9-1.9)	1.5 (0.9-2.7)	1.4 (1.0-2.0)	1.0 (0.9-1.2)
50-59		**1.2 (1.0-1.4)**	1.4 (0.6-3.1)	1.2 (1.0-1.5)	**3.5 (1.9-6.5)**	**2.4 (1.7-3.5)**	**2.0 (1.1-3.5)**	1.1 (0.7-1.6)	**1.4 (1.2-1.6)**
60-64		1.2 (0.9-1.6)	**3.9 (1.7-9.0)**	0.9 (0.6-1.2)	**4.1 (1.8-9.2)**	1.7 (1.0-3.1)	**3.7 (1.8-7.6)**	1.6 (0.9-3.0)	**1.5 (1.2-2.0)**
65-69		**1.4 (1.0-1.8)**	1.4 (0.5-4.0)	1.2 (0.9-1.7)	**4.3 (1.9-9.7)**	**2.8 (1.6-4.9)**	**4.9 (2.4-9.8)**	0.9 (0.4-1.9)	**1.9 (1.5-2.5)**
70-74		**1.9 (1.4-2.6)**	**3.9 (1.6-10.0)**	**1.9 (1.3-2.6)**	**6.4 (2.8-14.5)**	1.8 (0.9-3.4)	**8.2 (4.1-16.4)**	**2.0 (1.0-3.9)**	**2.1 (1.6-2.8)**
75-79		**1.9 (1.3-2.6)**	**4.6 (1.7-12.5)**	**2.7 (1.9-3.9)**	**8.2 (3.4-19.7)**	**3.5 (1.9-6.7)**	**16.4 (8.1-33.2)**	0.2 (0.0-1.4)	**2.5 (1.8-3.5)**
80-84		**2.2 (1.5-3.3)**	**6.6 (2.3-18.7)**	**2.4 (1.6-3.7)**	**7.4 (2.7-19.8)**	**3.7 (1.8-7.6)**	**15.1 (7.1-32)**	0.8 (0.2-2.9)	**2.1 (1.5-3.1)**
≥85		**4.0 (2.3-6.8)**	**17.3 (5.5-53.9)**	**6.6 (3.8-11.3)**	**7.2 (1.8-29.3)**	3.1 (1.0-9.9)	**15.2 (5.8-40.1)**	**3.4 (1.0-11.1)**	**3.0 (1.7-5.3)**
**Region of residence**									
West		1.0	1.0	1.0	1.0	1.0	1.0	1.0	1.0
Paris region		**1.4 (1.1-1.6)**	0.9 (0.4-2.0)	**2.5 (2.0-3.2)**	1.2 (0.7-2.3)	1.0 (0.7-1.5)	1.0 (0.7-1.6)	1.4 (0.9-2.3)	1.1 (0.9-1.3)
Eastern Parisian Basin		0.8 (0.6-1.0)	1.1 (0.8-1.4)	**0.8 (0.6-1.0)**	1.1 (0.6-2.1)	0.8 (0.6-1.2)	0.8 (0.5-1.2)	1.2 (0.7-2.0)	1.0 (0.9-1.2)
Western Parisian Basin		1.1 (0.8-1.4)	0.5 (0.2-1.7)	1.0 (0.7-1.5)	1.0 (0.5-2.2)	0.9 (0.5-1.5)	0.7 (0.4-1.3)	1.0 (0.5-1.9)	1.0 (0.8-1.2)
East		0.9 (0.7-1.1)	2.1 (0.9-4.9)	**1.5 (1.1-2.0)**	1.0 (0.4-2.1)	0.8 (0.5-1.4)	0.7 (0.4-1.2)	1.7 (1.0-2.9)	0.9 (0.7-1.1)
Mediterranean Basin		**2.2 (1.8-2.7)**	0.9 (0.4-2.2)	**2.7 (2.0-3.5)**	1.8 (1.0-3.3)	0.8 (0.5-1.3)	0.9 (0.5-1.4)	1.0 (0.6-1.8)	1.2 (1.0-1.4)
North		1.2 (0.9-1.4)	0.8 (0.3-2.1)	**1.4 (1.0-1.8)**	1.0 (0.5-2.1)	0.9 (0.6-1.4)	0.9 (0.5-1.5)	1.4 (0.8-2.4)	1.2 (1.0-1.4)
South-East		0.9 (0.7-1.2)	0.7 (0.3-1.9)	1.3 (1.0-1.8)	1.5 (0.8-3.0)	1.0 (0.6-1.5)	0.6 (0.3-1.0)	0.9 (0.5-1.6)	0.9 (0.7-1.1)
South-West		1.1 (0.9-1.4)	1.6 (0.7-3.8)	**2.3 (1.7-3.0)**	1.1 (0.5-2.2)	1.2 (0.8-1.8)	0.8 (0.5-1.4)	1.3 (0.7-2.2)	0.9 (0.7-1.1)
**Education**									
Upper tertiary level		1.0	1.0	1.0	1.0	1.0	1.0	1.0	1.0
No diploma		**2.3 (1.9-2.9)**	**64.6 (8.7-481.0)**	**3.3 (2.4-4.4)**	1.7 (0.8-3.9)	**1.9 (1.1-3.4)**	**15.5 (4.6-52.5)**	**3.0 (1.7-5.5)**	**1.9 (1.5-2.4)**
Primary school		**1.9 (1.5-2.3)**	**30.2 (4.1-224.2)**	**3.2 (2.4-4.2)**	**2.0 (1.1-3.7)**	**1.9 (1.2-3.1)**	**17.3 (5.4-55.2)**	**2.0 (1.1-3.6)**	**1.7 (1.4-2.1)**
Lower secondary level		1.1 (0.8-1.4)	8.8 (1.0-81.3)	**1.8 (1.3-2.5)**	1.2 (0.5-2.8)	1.2 (0.7-2.2)	**5.4 (1.4-20.4)**	1.7 (0.9-3.2)	1.2 (0.9-1.5)
Intermediate Secondary level		**1.4 (1.1-1.7)**	4.6 (0.6-37.1)	**1.7 (1.3-2.2)**	1.2 (0.7-2.3)	**1.6 (1.0-2.5)**	**7.8 (2.4-25.2)**	1.6 (1.0-2.8)	**1.4 (1.2-1.7)**
Upper Secondary level		**1.2 (0.9-1.5)**	5.9 (0.7-51.8)	**1.7 (1.2-2.3)**	0.9 (0.4-2.0)	1.0 (0.6-1.8)	**4.8 (1.3-16.8)**	1.2 (0.7-2.4)	1.2 (1.0-1.6)
Lower tertiary level		1.0 (0.8-1.2)	0.9 (0.1-13.8)	1.2 (0.9-1.6)	0.7 (0.3-1.5)	0.9 (0.5-1.5)	3.4 (0.9-11.9)	1.0 (0.6-1.8)	1.0 (0.8-1.2)
**Marital status**									
Married/in couple		1.0	1.0	1.0	1.0	1.0	1.0	1.0	1.0
Single		**1.4 (1.3-1.7)**	**3.1 (1.8-5.3)**	**1.6 (1.3-1.8)**	**1.6 (1.0-2.5)**	1.0 (0.7-1.6)	1.5 (0.9-2.4)	1.3 (0.8-2.2)	**1.2 (1.0-1.5)**
Divorced/separated		1.1 (0.8-1.3)	**3.3 (1.7-6.3)**	**1.4 (1.1-1.8)**	0.9 (0.4-1.7)	1.3 (0.9-1.7)	1.8 (1.2-2.7)	1.1 (0.8-1.6)	1.1 (1.0-1.3)
Widowed		1.0 (0.8-1.4)	**2.1 (1.1-4.2)**	0.8 (0.6-1.2)	0.9 (0.5-1.9)	0.9 (0.5-1.6)	1.0 (0.6-1.5)	1.2 (0.5-2.5)	1.0 (0.8-1.3)
**Geographic Origin**									
Metropolitan France		1.0	1.0	1.0	1.0	1.0	1.0	1.0	1.0
Foreign background		**1.8 (1.6-2.1)**	**13.6 (8.8-21.0)**	**1.9 (1.6-2.2)**	1.0 (0.6-1.7)	1.0 (0.7-1.4)	**1.7 (1.2-2.5)**	**1.5 (1.1 -2.2)**	**1.5 (1.3-1.7)**
**Occupation (active)**									
Yes		1.0	1.0	1.0	1.0	1.0	1.0	1.0	1.0
No		**1.2 (1.0-1.3)**	**1.8 (1.0-3.1)**	1.2 (1.0-1.4)	1.3 (0.8-2.1)	**1.4 (1.0-2.0)**	1.5 (0.9-2.3)	1.3 (0.9-1.9)	1.1 (0.9-1.2)
**Elixhauser score** (per point of score)		**0.8 (0.7-0.9)**	**1.9 (1.3-2.6)**	1.1 (1.0-1.3)	1.1 (0.8-1.5)	1.0 (0.7-1.3)	**1.2 (1.0-1.5)**	0.7 (0.4-1.0)	**1.2 (1.0-1.3)**
**(Un)complicated Hypertension**									
No		1.0	1.0	1.0	1.0	1.0	1.0	1.0	1.0
Yes		**0.6 (0.4-0.7)**	**0.3 (0.1-0.5)**	**0.7 (0.6-0.9)**	0.7 (0.4-1.3)	0.9 (0.6-1.4)	**0.4 (0.3-0.6)**	1.4 (0.8-2.6)	**0.7 (0.5-0.8)**
**Chronic pulmonary and pulm. circulation disorders**									
No		1.0	1.0	1.0	1.0	1.0	1.0	1.0	1.0
Yes		**0.6 (0.5-0.8)**	**0.4 (0.2-0.8)**	0.8 (0.6-1.1)	0.7 (0.4-1.5)	0.6 (0.4-1.1)	0.9 (0.6-1.5)	1.8 (1.0-3.3)	0.9 (0.7-1.1)
**Anxiety disorders**									
No		1.0	1.0	1.0	1.0	1.0	1.0	1.0	1.0
Yes		0.4 (0.2-1.0)	**7.1 (2.3-22.3)**	0.5 (0.2-1.3)	0.7 (0.1-5.4)	0.7 (0.2-2.9)	1.8 (0.6-5.2)	**<0.001**	1.0 (0.6-1.8)
**Migraine**									
No		1.0	1.0	1.0	1.0	1.0	1.0	1.0	1.0
Yes		**0.4 (0.3-0.6)**	0.8 (0.3-2.1)	**0.4 (0.3-0.7)**	0.2 (0.0-1.5)	1.4 (0.8-2.4)	1.2 (0.6-2.4)	0.9 (0.4-1.8)	1.0 (0.8-1.3)
**Bilateral blindness**									
No		1.0	1.0	1.0	1.0	1.0	1.0	1.0	1.0
Yes		0.7 (0.3-1.5)	**14.8 (7.1-32.9)**	1.8 (1.0-3.2)	0.6 (0.1-4.5)	1.4 (0.5-4.1)	**2.8 (1.3-5.9)**	0.7 (0.1-5.0)	1.4 (0.8-2.5)
**B**		**NON RESPONDERS**			**PARTIAL RESPONDERS**				
	**Complete and consistent (Reference)**	**Absent at the 2-month visit**	**Incapable of responding**	**Not willing to fill-in**	**> 2 missing physical and role items**	**> 2 missing mental items**	**> 2 missing physical and role items and > 2 missing mental items**	**Complete questionnaires with > 2 inconsistencies**	**Other (slightly) incomplete and inconsistent questionnaire**
		**OR (95% CI)**	**OR (95% CI)**	**OR (95% CI)**	**OR (95% CI)**	**OR (95% CI)**	**OR (95% CI)**	**OR (95% CI)**	**OR (95% CI)**
**Age**									
18-24	…	1.2 (1.0-1.5)	0.2 (0.0-1.9)	0.8 (0.6-1.1)	0.8 (0.4-1.6)	0.7 (0.4-1.2)	<0.001	1.6 (0.9-3)	**0.8 (0.6-0.9)**
25-39		1.0	1.0	1.0	1.0	1.0	1.0	1.0	1.0
40-49		1.0 (0.8-1.2)	**3.1 (1.5-6.8)**	1.2 (0.9-1.4)	1.5 (0.9-2.6)	**1.9 (1.4-2.7)**	**3.1 (1.7-5.5)**	1.0 (0.6-1.7)	**1.2 (1.0-1.4)**
50-59		**1.5 (1.2-1.8)**	**2.9 (1.2-7.1)**	**1.5 (1.2-2.0)**	**2.2 (1.2-3.9)**	**2.9 (2.0-4.3)**	**5.3 (2.9-9.6)**	1.6 (0.9-2.7)	**1.4 (1.2-1.7)**
60-64		**1.5 (1.1-2.0)**	1.5 (0.5-4.4)	**1.8 (1.3-2.4)**	**2.8 (1.4-5.6)**	**4.1 (2.6-6.6)**	**9.7 (5.1-18.4)**	1.1 (0.5-2.3)	**1.6 (1.3-2.0)**
65-69		**2.3 (1.7-3.0)**	1.2 (0.4-3.7)	**2.6 (1.9-3.5)**	**4.9 (2.5-9.7)**	**4.1 (2.5-6.9)**	**11.7 (6.1-22.4)**	1.6 (0.8-3.3)	**1.9 (1.5-2.4)**
70-74		**2.8 (2.1-3.8)**	0.9 (0.3-3.0)	**3.2 (2.3-4.3)**	**5.9 (3.0-11.7)**	**5.4 (3.2-9.1)**	**20.3 (10.8-38.4)**	1.5 (0.7-3.2)	**2.4 (1.9-3.1)**
75-79		**3.5 (2.5-4.9)**	2.6 (0.9-7.5)	**4.8 (3.4-6.6)**	**6.4 (3.1-13.4)**	**6.9 (4.0-11.9)**	**26.3 (13.7-50.5)**	**2.7 (1.3-5.7)**	**2.5 (1.9-3.3)**
80-84		**5.7 (3.9-8.3)**	**11.3 (4.2-30.0)**	**6.1 (4.1-9.0)**	**6.2 (2.5-15.0)**	**6.4 (3.2-12.6)**	**39.9 (20.1-79.3)**	**3.5 (1.5-8.2)**	**3.2 (2.3-4.5)**
≥85		**3.3 (2.0-5.3)**	**5.5 (1.8-16.9)**	**4.5 (2.8-7.2)**	**8.8 (3.5-22.2)**	**4.7 (2.0-11.1)**	**17.5 (7.7-39.5)**	1.1 (0.2-4.9)	**1.8 (1.2-2.8)**
**Education**									
Upper tertiary level	…	1.0	1.0	1.0	1.0	1.0	1.0	1.0	1.0
No diploma		**2.4 (1.7-3.4)**	**11.6 (1.5-89.6)**	**1.8 (1.2-2.6)**	2.7 (1.0-7.5)	1.7 (0.9-3.1)	**3.4 (1.2-10.0)**	**4.0 (1.3-12.0)**	**2.5 (1.9-3.4)**
Primary school		**1.6 (1.1-2.2)**	4.7 (0.6-36.1)	**1.8 (1.2-2.6)**	2.2 (0.8-5.9)	1.5 (0.8-2.6)	**3.3 (1.2-9.2)**	**3.6 (1.2-10.5)**	**2.4 (1.8-3.1)**
Lower secondary level		1.1 (0.7-1.5)	0.6 (0.0-6.5)	0.9 (0.6-1.3)	1.3 (0.5-3.7)	1.1 (0.6-2.0)	1.7 (0.6-5.1)	2.0 (0.6-6.1)	**1.6 (1.2-2.1)**
Intermediate Secondary level		0.9 (0.6-1.2)	0.5 (0.1-4.7)	0.8 (0.6-1.2)	1.5 (0.6-3.8)	1.4 (0.8-2.4)	1.4 (0.5-4.1)	2.1 (0.7-6.1)	**1.6 (1.3-2.1)**
Upper Secondary level		1.1 (0.8-1.5)	0.5 (0.0-6.0)	1.0 (0.7-1.5)	0.9 (0.3-2.6)	0.9 (0.5-1.7)	2.1 (0.7-5.9)	1.5 (0.5-4.5)	**1.5 (1.1-1.9)**
Lower tertiary level		0.9 (0.7-1.3)	0.7 (0.1-7.0)	1.0 (0.7-1.4)	1.1 (0.4-3.0)	0.9 (0.5-1.5)	1.1 (0.4-3.2)	1.4 (0.5-4.2)	**1.4 (1.1-1.8)**
**Marital status**									
Married/in couple		1.0	1.0	1.0	1.0	1.0	1.0	1.0	1.0
Single		**1.4 (1.2-1.6)**	1.1 (0.6-2.2)	1.1 (0.9-1.3)	1.3 (0.8-2.0)	1.1 (0.8-1.5)	**1.6 (1.1-2.3)**	0.8 (0.5-1.3)	1.1 (1.0-1.2)
Divorced/separated		**1.6 (1.3-1.9)**	1.0 (0.5-2.2)	**1.5 (1.2-1.9)**	1.2 (0.7-2.0)	**1.7 (1.3-2.3)**	1.2 (0.8-1.8)	1.1 (0.7-1.8)	**1.2 (1.0-1.4)**
Widowed		**1.5 (1.3-1.9)**	1.4 (0.8-2.4)	**1.3 (1.1-1.6)**	1.1 (0.7-1.7)	1.2 (0.9-1.7)	1.3 (1.0-1.7)	0.9 (0.6-1.6)	**1.2 (1.0-1.4)**
**Occupation (active)**	…								
Yes		1.0	1.0	1.0	1.0	1.0	1.0	1.0	1.0
No		1.1 (1.0-1.3)	**7.7 (3.6-16.6)**	1.1 (0.9-1.3)	**1.5 (1.0-2.2)**	1.0 (0.8-1.3)	**1.5 (1.1-2.2)**	**1.5 (1.1-2.2)**	**1.1 (1.0-1.2)**
**Income** (10,000 €/year/household unit)		**0.9 (0.8-0.9)**	0.9 (0.7-1.1)	**0.8 (0.8-0.9)**	0.9 (0.8-1.1)	1.0 (0.9-1.1)	**0.7 (0.6-0.9)**	0.9 (0.8-1.1)	1.0 (1.0-1.0)
**Elixhauser score** (per point of score)		**0.9 (0.8-1.0)**	1.2 (0.8-1.6)	1.0 (0.8-1.1)	0.9 (0.7-1.2)	1.0 (0.9-1.3)	1.0 (0.8-1.2)	**1.2 (1.0-1.6)**	1.0 (0.9-1.1)
**(Un)complicated Hypertension**									
No		1.0	1.0	1.0	1.0	1.0	1.0	1.0	1.0
Yes	…	**0.7 (0.5-0.8)**	1.0 (0.6-1.6)	0.9 (0.7-1.1)	1.2 (0.8-1.9)	0.7 (0.5-1.0)	1.1 (0.8-1.4)	0.9 (0.6-1.4)	1.0 (0.8-1.2)
**Paralysis**									
No		1.0	1.0	1.0	1.0	1.0	1.0	1.0	1.0
Yes		**2.7 (1.1-6.4)**	**7.0 (2.1-23.3)**	1.0 (0.3-3.1)	**<0.001**	3.0 (0.9-9.6)	1.5 (0.4-5.5)	**<0.001**	1.2 (0.5-2.8)
**Other neurological disorders**									
No		1.0	1.0	1.0	1.0	1.0	1.0	1.0	1.0
Yes		1.2 (0.6-2.2)	**3.4 (1.2-9.4)**	1.1 (0.6-2.2)	1.9 (0.6-6.5)	1.9 (0.8-4.4)	**2.4 (10-5.4)**	1.0 (0.2-4.1)	0.9 (0.5-1.5)
**Diabetes**									
No		1.0	1.0	1.0	1.0	1.0	1.0	1.0	1.0
Yes		**1.4 (1.0-1.9)**	**2.1 (1.1-4.0)**	**1.6 (1.2-2.2)**	1.0 (0.5-2.0)	0.7 (0.4-1.3)	1.5 (1.0-2.2)	1.0 (0.5-2.1)	1.1 (0.8-1.4)
**Migraine**									
No		1.0	1.0	1.0	1.0	1.0	1.0	1.0	1.0
Yes		**0.6 (0.5-0.8)**	0.8 (0.3-1.8)	**0.6 (0.5-0.9)**	1.0 (0.6-1.7)	0.8 (0.6-1.3)	1.1 (0.7-1.7)	0.9 (0.5-1.5)	1.0 (0.9-1.2)
**Sleeping disorders**									
No		1.0	1.0	1.0	1.0	1.0	1.0	1.0	1.0
Yes		**0.5 (0.4-0.8)**	0.8 (0.3-2.0)	**0.6 (0.4-0.9)**	1.0 (0.5-2.0)	0.8 (0.5-1.5)	0.8 (0.5-1.3)	**1.9 (1.1-3.4)**	0.8 (0.6-1.0)
**Visual deficiency**									
No		1.0	1.0	1.0	1.0	1.0	1.0	1.0	1.0
Yes		0.9 (0.8-1.1)	0.6 (0.4-1.0)	**0.8 (0.7-1.0)**	0.6 (0.4-1.0)	0.9 (0.7-1.2)	1.0 (0.7-1.4)	0.8 (0.5-1.1)	1.0 (0.9-1.1)

Statistically significant odds ratios are in bold. Panel A: men, Panel B: women.

### Bias in HRQoL estimation due to non- and partial responses

The scores computed using standard rules in responders only were compared to those obtained after imputation of all missing values: the differences appeared modest overall (<0.25 standard deviation) for all dimensions. “Standard results”, considering only responders, overestimated scores of physical and role dimensions (PF, RP, RE) and underestimated those of “mental” dimensions (GH, VT, SF MH). Also, the biases (of either direction) generally increased with age. Table 4 shows the detailed scores obtained for two informative and contrasted dimensions (physical functioning and general health) in the different groups of responders considered. For the PF dimension (and other role dimensions, not shown), scores for all partial responders were generally slightly underestimated by using standard rules. Exceptions to this were at older ages and for inconsistent responses where the impact on estimates of non-differential information biases due to the use of standard rules appeared to be the opposite i.e., causing large overestimation. A similar change in the direction of bias was observed with age for non-responders: “absent” and “declining to fill-in” subjects had generally lower scores than complete and consistent responders at younger ages, and much higher scores at older ages. The magnitude of bias was large in some groups of partial and especially non-responders. However, the opposite directions of selection biases and non-differential information biases caused the overall differences to be small. For the GH dimension (and most other mental dimensions, not shown), biases were smaller, had generally a U-shaped relationship with age; they also showed the same sort of opposites compensating effects on the overall differences.

**Table 4:** Scores computed using standard rules and/or after imputation of the missing values

		**Non responders**			**Partial responders**					**All**		
	**Complete and consistent**	**Absent at the 2-month visit**	**Incapable of responding**	**Not willing to fill-in**	**> 2 missing physical and role items**	**> 2 missing mental items (a)**	**> 2 missing physical and role items and > 2 missing mental items**	**Complete questionnaires with > 2 inconsistancies**	**Other (slightly) incomplete and inconsistent questionnaire**			
	**Mean score**	**Bias 1**	**Bias 1**	**Bias 1**	**Bias 2**	**Bias 2**	**Bias 2**	**Bias 2**	**Bias 2**	**Mean (SD) score (using standard scoring rules)**	**Mean (SD) score (after imputation)**	**Bias 2**
**A**												
<60 yrs.	92.01	-2.78	-11.93	-3.92	3.75	0.00	6.62	-8.86	-0.91	91.06 (15.96)	90.18 (16.86)	-0.88
60 - 64 yrs	82.28	-6.08	-12.97	-8.59	2.89	0.00	3.40	-11.97	-0.83	80.90 (21.97)	79.01 (21.27)	-1.88
65 - 69 yrs.	77.03	-7.58	-13.78	-8.26	-1.91	0.00	5.57	-24.46	-1.06	75.63 (23.11)	73.10 (22.00)	-2.52
70 - 74 yrs.	69.69	-3.42	-12.59	-5.09	0.48	0.00	7.56	-7.20	-1.36	68.26 (26.25)	66.93 (23.64)	-1.33
75 - 79 yrs	64.21	-3.26	-10.14	-5.73	0.79	0.00	5.93	9.72	-0.60	61.66 (27.73)	60.78 (24.19)	-0.88
80 - 84 yrs	52.74	1.10	-4.48	0.12	-0.54	0.00	7.49	3.32	-0.66	51.18 (29.74)	52.11 (24.97)	0.93
≥85 yrs.	37.91	9.03	-0.65	7.91	-9.15	0.00	7.13	0.00	0.68	40.74 (31.87)	43.65 (25.69)	2.91
**B**												
<60 yrs.	67.60	3.70	-8.30	1.59	0.00	-0.26	0.15	0.00	0.00	67.64 (19.99)	68.26 (20.01)	0.62
60 - 64 yrs	61.32	2.65	-8.03	-2.17	0.00	0.34	-1.29	0.00	-0.01	60.68 (20.90)	60.81 (20.97)	0.13
65 - 69 yrs.	56.79	2.03	-9.06	0.34	0.00	-0.67	-1.01	0.00	0.01	56.60 (21.54)	56.82 (21.39)	0.23
70 - 74 yrs.	54.37	3.19	-8.21	0.23	0.00	0.00	-0.59	0.00	-0.17	53.49 (22.08)	54.10 (21.84)	0.61
75 - 79 yrs	52.35	3.18	-7.97	-0.88	0.00	-0.77	0.34	0.00	-0.1	51.73 (22.01)	52.14 (21.69)	0.41
80 - 84 yrs	48.07	3.45	-2.76	-0.04	0.00	0.00	-0.34	0.00	-0.13	46.62 (23.56)	47.71 (22.78)	1.09
≥85 yrs.	44.94	4.25	-7.78	1.71	0.00	0.00	-1.17	0.00	-0.39	47.94 (24.20)	47.28 (22.84)	-0.67

Panel A: Physical functioning, Panel B: General Health.

Bias 1: mean score after imputation – mean score of the complete-consistent group.

Bias 2: mean score after imputation – mean score obtained using standard scoring rules.

(a) Subjects with incomplete questionnaires only for mental dimensions by definition had no missing values for any of PF items.

(b) Subjects with incomplete questionnaires only for physical and role dimensions by definition had no missing values for any of GH items; Subjects with inconsistent questionnaire had no inconsistencies recorded for the GH dimension (see Table 3 for the list of inconsistencies).

## Discussion

As HRQoL measures are increasingly used in the general population, it is important to consider various forms of, and reasons for, non-optimal assessment and the extent of potential resulting biases. This study is the first to consider, comprehensively and simultaneously, non response, and incomplete and inconsistent responses to a widely used HRQoL, the SF-36, and their consequences in terms of the validity of estimates, in a general population setting.

Only a half of eligible subjects were found to provide an optimal (complete and consistent) measurement of HRQoL. This proportion could be increased to 66% by accepting sub-optimal (slightly incomplete and inconsistent) questionnaires, specifically those questionnaires that could be reasonably and easily handled using standard rules for managing missing data i.e. the “half item” rule and personal mean score [11,20] after identifying inconsistencies. Note, however, that such procedures are not widely used in practice despite the fact that they are straightforward and simple to implement. Therefore, for one third of the general adult population that could be assessed for common health questions by face-to-face interview, self assessment of HRQoL using a standardized questionnaire was unsatisfactory. The three groups of subjects we identified with inadequate measurement were of different sizes: 25% of all eligible subjects were non-responders, 6% were poor or very partial responders and 2% inconsistent responders. However, these three groups shared similar socio-demographic determinants. Indeed, several common factors were found associated with both non- and partial response to the SF-36, the strongest being age and educational level. HRQoL is predictive of mortality [21] and validly reflects the cumulative burden of chronic diseases and disabilities. Clearly, aging populations have been, and will be, targeted for HRQoL studies [22]. This study confirms problems of measurement of HRQoL in the elderly population, with an increased risk of all of non-, partial and inconsistent response after age 50 years. Among men and women aged 75 to 79 years, the proportions of inadequate measurement were about 50% and 55%, respectively, raising serious concerns about the use of a standard “generic” instruments (as is the SF-36) in such (healthy) older populations. Higher rates of missing items in HRQoL questionnaires have already been reported in elderly populations [7,23-28], but this problem has generally been minimized or resolved by minor adaptations of questionnaires or by interviewer administration [5,24,29]. Non responses (missing forms) for HRQoL measures has been less specifically investigated in relation to age, although this issue introduces a major selection bias [5]. However, the findings we report are supported by previous studies showing high non-response rates for elderly subjects to mailed surveys [30-33]. Educational level, marital status and other socio-economic characteristics have less often been considered than age in relation to missing items or non-participation in previous studies of HRQoL instruments. Nevertheless, the evidence available is consistent with the result that subjects with low educational level, foreign origin, low economic status and who are isolated (especially divorced and widowed) are at increased risk of having missing items in HRQoL questionnaires [7,27] and of non participation in mailed surveys [32,33]. In the same way as for HRQoL measurement in elderly populations, precautions may be required when measuring HRQoL in groups of subjects less well-educated and well-integrated into western societies.

The relationships between morbid conditions and non- and partial responses observed appeared more complex than expected: some conditions were associated with increased, and others with decreased, partial and non-response rates. Despite the low power of this study for some important but uncommon conditions, as shown in the wide confidence intervals around odds ratios (Table 3), and possible type I error due to testing almost 30 such conditions, a consistent pattern emerged from the data: this pattern suggests that subjects with minor somatic and psychological disorders (e.g. hypertension, anxiety and migraine) are more likely to accept HRQoL measurement than both “healthy” and more seriously affected subjects. Possibly, these subjects whose condition is closely related to impaired HRQoL (i.e. whose expression is mostly decreased HRQoL) find its assessment particularly relevant and are therefore more likely to respond and to do so more meticulously than “average” subjects. However, this behavior, which has not been previously reported, requires further confirmation and also more rigorous analysis in terms of its potential contribution to bias in HRQoL measurements.

Using a multiple imputation method to provide the best corrected estimates of HRQoL in the sample studied, it was possible to assess and quantify the impact of non- and partial responses on the validity of HRQoL estimates. The magnitude of the biases was large in several groups of partial responders and especially non-responders. This confirms the “missing not random” process of missing information in HRQoL, to use the terminology coined by Little and Rubin [34]. These biases, including selection biases [35] but also non-differential information biases [36], should be carefully considered. Non-responders in epidemiological studies have long been recognized to have an impact on the validity of the results. Our study evidenced several groups of non-responders to HRQoL questionnaires having different and sometimes opposite impacts on the estimates. This argues for a differentiated approach taking their different causes and/or mechanisms into account. Similarly, non-differential information biases, resulting from partial or inconsistent responses to HRQoL questionnaires, did not appear to be negligible. These biases were especially large for the subgroup of subjects with inconsistent responses, which are seldom examined in standard practice. Although in this study we observed that biases may run in opposite directions and partially neutralize each other, this may of course not be always the case and therefore a careful analysis of the impact of each is required. This issue is particularly pertinent for HRQoL investigations in certain populations: the elderly, and deprived or frail groups. No simple general rule can be given to predict the impact on HRQoL estimation of missing data associated with the various different processes. We therefore strongly recommend using missing value methods such as multiple imputation to evaluate the consequences systematically [4-9,11].

In conclusion, this empirical study confirms serious problems with HRQoL measurement in the general population due to missing data (both items and forms), especially in elderly, educationally and socio-economically deprived, foreign and isolated groups. Missing data methods and imputation techniques, which are increasingly implemented in standard software packages (SAS, SPSS, etc.), appear to be useful for quantifying potential biases and are therefore recommended to evaluate HRQoL estimates systematically and, if necessary, correct for the resulting biases.

## Abbreviations

BP: Bodily pain; GH: General health perceptions; HRQoL: Health related Quality of life; ICD-10: International classification of diseases, 10th revision; INSEE: French National Institute of Statistics and Economic Studies; IQOLA: International Quality of Life Assessment; MH: Mental health; PF: Physical functioning; RE: Role limitation relating to mental health; RP: Role limitations relating to physical health; SF: Social functioning; SF-36: Medical Outcome Study 36-item short-form health survey; VT: Vitality

## Competing interests

The authors declare that they have no competing interests.

## Authors’ contributions

JC conceived the study, participated in its design and drafted the manuscript. LQ participated in the design of the study and performed the statistical analysis. JC provided administrative, technical and logistic support. All authors read and approved the final manuscript.

## Supplementary Material

Additional file 1: Table S1 Most frequently reported morbidities by subjects eligible for HRQoL measurement (n = 30,996). Figures are numbers (percentages). Click here for file
