# Supplementary material for: Non response, incomplete and inconsistent responses to self-administered health-related quality of life measures in the general population: patterns, determinants and impact on the validity of estimates — a population-based study in France using the MOS SF-36
Source: Health Qual Life Outcomes. 2013 Mar 13;11:44. doi: 10.1186/1477-7525-11-44 (PMC3606448; doi:10.1186/1477-7525-11-44)
Supplement: Additional file 1: Table S1 — Most frequently reported morbidities by subjects eligible for HRQoL measurement (n = 30,996). Figures are numbers (percentages). [file 1477-7525-11-44-S1.docx]

Supplementary Table 1. Most frequently reported morbidities by subjects eligible for HRQoL measurement (n=30,996). Figures are numbers (percentages)

Lymphoma and solid tumor without metastasis 509 (1.7)

Metastatic cancer 88 (0.3)

Obesity (body mass index >30) 3013 (9.8)

Diabetes 1181 (3.8)

Hypothyroidism 159 (0.5)

Parkinson disease 55 (0.2)

Migraine 1450 (4.7)

Paralysis 114 (0.4)

Other neurological disorders 223 (0.7)

Anxiety disorders 327 (1.1)

Depression 1120 (3.6)

Memory deficiency 141 (0.5)

Sleeping disorders 633 (2.1)

Cataract 351 (1.1)

Glaucoma 346 (1.1)

Bilateral blindness 230 (0.7)

Visual deficiency 20003 (65.0)

Hearing deficiency 3507 (11.4)

Congestive heart failure 74 (0.2)

Cardiac arrhythmias 480 (1.6)

Valvular disease 88 (0.3)

Peripheral vascular disorders 292 (0.9)

(Un)complicated Hypertension 3804 (12.4)

Chronic pulmonary and pulmonary circulation disorders 1938 (6.3)

Liver disease 96 (0.3)

Peptic ulcer disease (excluding bleeding) 138 (0.4)

Renal failure 79 (0.3)

Rheumatoid arthritis and collagen vascular disease 111 (0.4)
